# CT severity score in COVID-19 patients, assessment of performance in triage and outcome prediction: a comparative study of different methods

**DOI:** 10.1186/s43055-022-00781-5

**Published:** 2022-05-18

**Authors:** Alireza Almasi Nokiani, Razieh Shahnazari, Mohammad Amin Abbasi, Farshad Divsalar, Marzieh Bayazidi, Azadeh Sadatnaseri

**Affiliations:** 1 Department of Radiology, Firoozabadi Hospital, Iran University of Medical Sciences, Tehran, Iran; 2 Firoozabadi Clinical Research Development Unit (FCRDU), Iran University of Medical Sciences, Tehran, Iran; 3 Present Address: Department of Cardiology, Sina Hospital, Tehran University of Medical Sciences, Imam Komeini St., Tehran, 1136746911 Iran

**Keywords:** COVID-19, CT quantification, Triage, Prognosis, ROC curve, Heart failure, Interrater reliability, Computed tomography, Intraclass correlation coefficient, CT severity score

## Abstract

**Background:**

Lung involvement in COVID-19 can be quantified by chest CT scan with some triage and prognostication value. Optimizing initial triage of patients could help decrease adverse health impacts of the disease through better clinical management. At least 6 CT severity score (CTSS) systems have been proposed. We aimed to evaluate triage and prognostication performance of seven different CTSSs, including one proposed by ourselves, in hospitalized COVID-19 patients diagnosed by positive polymerase chain reaction (PCR).

**Results:**

After exclusion of 14 heart failure and significant preexisting pulmonary disease patients, 96 COVID-19, PCR-positive patients were included into our retrospective study, admitted from February 20, 2020, to July 22. Their mean age was 63.6 ± 17.4 years (range 21–88, median 67). Fifty-seven (59.4%) were men, and 39 (40.6%) were women. All CTSSs showed good interrater reliability as calculated intraclass correlation coefficients (ICCs) between two radiologists were 0.764–0.837. Those CTSSs with more numerous segmentations showed the best ICCs. As judged by area under curve (AUC) for each receiver operator characteristic (ROC) curve, only three CTSSs showed acceptable AUCs (AUC = 0.7) for triage of severe/critical patients. All CTSSs showed acceptable AUCs for prognostication (AUCs = 0.76–0.79). Calculated AUCs for different CTSSs were not significantly different for triage and for prediction of severe/critical disease, but some difference was shown for prediction of critical disease.

**Conclusions:**

Men are probably affected more frequently than women by COVID-19. Quantification of lung disease in COVID-19 is a readily available and easy tool to be used in triage and prognostication, but we do not advocate its use in heart failure or chronic respiratory disease patients. The scoring systems with more numerous segmentations are recommended if any future imaging for comparison is contemplated. CTSS performance in triage was much lower than earlier reports, and only three CTSSs showed acceptable AUCs in this regard. CTSS performed better for prognostic purposes than for triage as all 7 CTSSs showed acceptable AUCs in both types of prognostic ROC curves. There is not much difference among performance of different CTSSs.

## Background

Because of the primary involvement of the respiratory system, chest computed tomography (CT) is strongly recommended in suspected COVID-19 cases, for both initial evaluation and follow-up [1]. Lung involvement in COVID-19 can be quantified by chest CT with triage and prognostication value [1–11]. Optimizing initial triage of patients could help to decrease adverse health impact of the disease through better clinical management, efficient prioritization of cases and timely discharge of admitted patients [11]. At least six scoring systems using chest CT have been proposed to quantify lung involvement in COVID-19 which are summarized in Table 1 [1–10]. We use the term CT severity score (CTSS) to refer to them with numbers 1–7 to refer to a specific scoring system. We introduced STSS7 for possible implementation in triage and prognostication. We aimed to determine the value of CTSSs in making decisions about the intensity of the treatment of respiratory failure (triage) and predicting the risk of development of severe/critical disease in the course of COVID-19 (prognostication). Comparison of different CTSSs was also done.

**Table 1:** Seven proposed COVID-19 CT severity score systems

CTSSs	Segmentation	Severity Score for each segment	Maximum Score
CTSS1 [2, 3]	Three zones in each lung are divided by carina and lower pulmonary vein	1–4 according to percentage of involvement (< 25, 25–49, 50–74, > 75)	24
CTSS2 [4]	The same zonal concept as CTSS1 with additional division of each zone into anterior and posterior regions divided by midpoint of diaphragm antero-posteriorly	1–4 according to percentage of involvement (< 25, 25–49, 50–74, > 75)	48
CTSS3 [5, 6]	Five anatomic lobes of the lungs	1–4 according to percentage of involvement (< 25, 25–49, 50–74, > 75)	20
CTSS4 [7, 8]	Five anatomic lobes of the lungs	1–5 according to percentage of involvement (< 5, 5–25, 25–49, 50–74, > 75)	25
CTSS5 [9]	Five anatomic lobes of the lungs	1–4 according to the diameter of the largest lesion in each lobe (< 1 cm, 1-3 cm, > 3 cm up to 50% of the lobe, > 50% of a lobe	20
CTSS6 [10]	18 anatomic segments of the lung with an additional division of apico-posterior segment of the left upper lobe into apical and posterior divisions and anteromedial segment of the left lower lobe into anterior and medial segments	No involvement = 0 < 50% involvement = 1 ≥ 50% involvement = 2	40
CTSS7 [current authors]	Five anatomic lobes of the lungs with additional consideration of the lingula as a separate lobe	1–5 according to percentage of involvement (< 5, 5–25, 25–49, 50–74, > 75)	30

Xie and colleagues used a CTSS based on dividing the lungs into upper, middle and lower zones, and each scored 0–4 according to percentage of involvement (CTSS1) [2]. They stated elsewhere that mean CTSS1 was significantly higher in severe/critical group than in mild/moderate group of patients (12.86 vs 5.34) [3]. Zhou and co-workers used a CTSS with the same zonal concept, further dividing each zone into anterior and posterior divisions with maximum 48 scores (CTSS2) [4]. There was no performance report. Chung and colleagues scored each of the five lung lobes by percentage of involvement from 0 to 4. CTSS was the sum of the five lobe scores, with a maximum of 20 (CTSS3) [5]. Li and colleagues implemented CTSS3 and reported an intraclass correlation coefficient (ICC) of 0.976 between two observers and area under the curve (AUC) of 0.918 for receiver operator characteristic (ROC) curve to diagnose severe/critical disease; the CTSS cutoff point of 7.5 had 82.6% sensitivity and 100% specificity [6]. Other researchers used another CTSS. Each of the 5 lung lobes was visually scored from 0 to 5 as: 0, no involvement; 1, < 5%; 2, 5–25%; 3, 26–49%; 4, 50–75%; and 5, > 75% involvement. Maximum total score was 25 (CTSS4) [7, 8]. They reported no ROC curve or cutoff point. Xiong and co-workers assessed each lobe for opacification and lesion size with a maximum sum of 20 (CTSS5) [9]. Yang and colleagues developed another CTSS in which the 18 segments of the lung were divided into 20 regions. The lung opacities in all the 20 lung regions were evaluated on chest CT using a system attributing scores of 0, 1 and 2 according to the absence or presence of 50% or more segmental opacification with a maximum of 40 (CTSS6). Interrater reliability for CTSS6 was excellent (ICC = 0.936). The area under the ROC curve for diagnosing patients in severe/critical group was 0.892 (95% confidence interval: 0.814–0.944). Optimal CTSS threshold for identifying severe/critical patients was 19.5, with 83.3% sensitivity and 94% specificity. The interrater reliability for CTSS6 was excellent (ICC_median_ = 0.925, ICC_mean_ = 0.936) [10]. We propose another CTSS which is almost the same as CTSS4, but considers lingula as a separate lobe (CTSS7) with a maximum score of 30.

## Methods

### Patients

Our institutional review board waived requirement to obtain written informed consent for this retrospective study which evaluated de-identified data and involved no potential risk for patients. To avert any potential breach of confidentiality, no link between the patients and the researchers was made available.

We enrolled patients with COVID-19 referred to Firoozabadi hospital, Tehran, Iran, from February 20, 2020, to July 22. The diagnosis was based on positive results of reverse-transcriptase polymerase chain reaction (RT-PCR) assay of nasal and pharyngeal swab specimens at any time during hospitalization. Exclusion criteria were significant cardiopulmonary comorbidity, defined as cardiothoracic ratio > 60% on CT topogram image [12] and diameter ratios of central branches of pulmonary artery to corresponding bronchi > 2 [13, 14] or preexisting pulmonary disease involving more than 30% of the lungs, diagnosed subjectively by visual assessment of the same CT images by the radiologist (AA). Patients that did not have any CT examination in our hospital were also excluded.

We retrospectively collected clinical and laboratory data from the hospital information system (HIS), including disease severity at presentation, severity in the most severe disease period, final outcome (death or discharge), place of hospital admission (ward or ICU), state of intubation and any comorbidity.

Severity of the disease was decided by the information derived from patients’ records as presented in Table 1 [15]. For less complexity when the exact required data were not available, we regarded those who had undergone tracheal intubation or had died from the disease as critical.

### Image acquisition

Chest CT imaging was performed by a 16-detector CT scanner (Emotion; Siemens; Germany). All patients were examined in supine position. CT images were then acquired during a single inspiratory breath-hold. The scanning range was from the apex of lung to costophrenic angle.

CT scan parameters: X-ray tube parameters—110KVp, 45–60 effective mAs; rotation time—0.6 s; collimation- 16 × 1.2; pitch—1.5; section thickness—5 mm; reconstruction interval—5 mm with B70 sharp convolution kernel; additional reconstructions at slice thickness; and reconstruction interval of 1.5 mm with B70 and B31 convolution kernels, were also made to generate lung and mediastinal windows, respectively. Lung window images were viewed at a width/level of 1200/-600 and mediastinal window images at 350/50 window settings.

### Image interpretation

Two radiologists with 17 and 3 years of experience (AAN and RSh, respectively) blinded to clinical data reviewed CT images of all the patients independently and scored each patient’s images according to each of the 7 scoring systems mentioned in the introduction section (Table 2). They viewed images on hospital PACS (Marco PACS Version 2.0.0.0) and resorted to multiplanar reconstruction (MPR) whenever needed. We took into account 11 of 14 imaging features defined in a previous study [16]: ground-glass opacity (GGO), consolidation, mixed GGO and consolidation, centrilobular nodules, architectural distortion, tree-in-bud, bronchial wall thickening, reticulation, subpleural bands, traction bronchiectasis and vascular enlargement in the lesion. Other relevant pathological findings such as enlarged heart, other pulmonary parenchymal disease such as cavities and emphysema, pleural effusion and mediastinal lymph nodes were also recorded.

**Table 2:** Clinical severity of COVID-19

Measured indicator/severity^a^	Mild	Moderate	Severe	Critical
Respiratory rate	≥ 24	≥ 30	–	–
SPO_2_	≥ 93	93 > SPO_2_ ≥ 90	89 > SPO_2_ ≥ 85	< 85^b^
Respiratory distress	None	None	Mild to moderate	Severe^c^
Blood pressure	–	–	–	< 90/60

^a^Presence of any of the severity indicators of the more severe group places the patient in the more severe group

^b^Despite high-flow O_2_ administration

^c^Nasal flaring, air hunger, intercostal retraction, subcostal retraction

### Statistical analysis

All statistical analyses were done using SPSS 26.0 software (IBM, Armonk, NY), excluding comparison of ROC curves AUCs and selection of cutoff points which were conducted by MedCalc statistical software version 19.9.4.0. *P* < 0.05 was considered statistically significant. Statistical analysis was performed by AAN. Quantitative data were expressed as mean ± standard deviation and/or median. Comparison of means was performed by independent sample t test for two means and ANOVA test for more than two means [17]. Interrater reliability was evaluated using intraclass correlation coefficient (ICCs) for CTSSs. ICC estimates and their 95% confidence intervals (CI) were calculated based on a two-way random model, single measurement form and absolute agreement type (ICC_1,1_ with absolute agreement) [18]. ICCs were classified as follows: poor reliability < 0.5; moderate reliability, 0.5–0.74; good reliability, 0.75–0.89; and excellent reliability, 0.9–1.0) [19]. ROC curve analysis was performed on the averages of reported CTSSs by the two raters for each CTSS to calculate AUC for diagnosing severe/critical COVID-19 at the time of hospital admission (for triage). Then, AUCs were classified unsatisfactory if AUC < 0.7, acceptable if 0.7 ≤ AUC < 0.8, excellent if 0.8 ≤ AUC < 0.9 and outstanding if AUC ≥ 0.9 [20]. The best threshold, specificity and sensitivity for the CTSSs were calculated. We chose best thresholds according to Youden index method which is choosing the threshold producing the largest Youden Index (sensitivity + specificity − 1) [21]. The AUCs for the ROC curves were compared pairwise by the z test.

The same statistical procedure was applied to the CTSSs for predicting severe/critical disease at peak disease severity and also for predicting critical disease at peak severity (for prognostication).

## Results

**Fig. 1:**
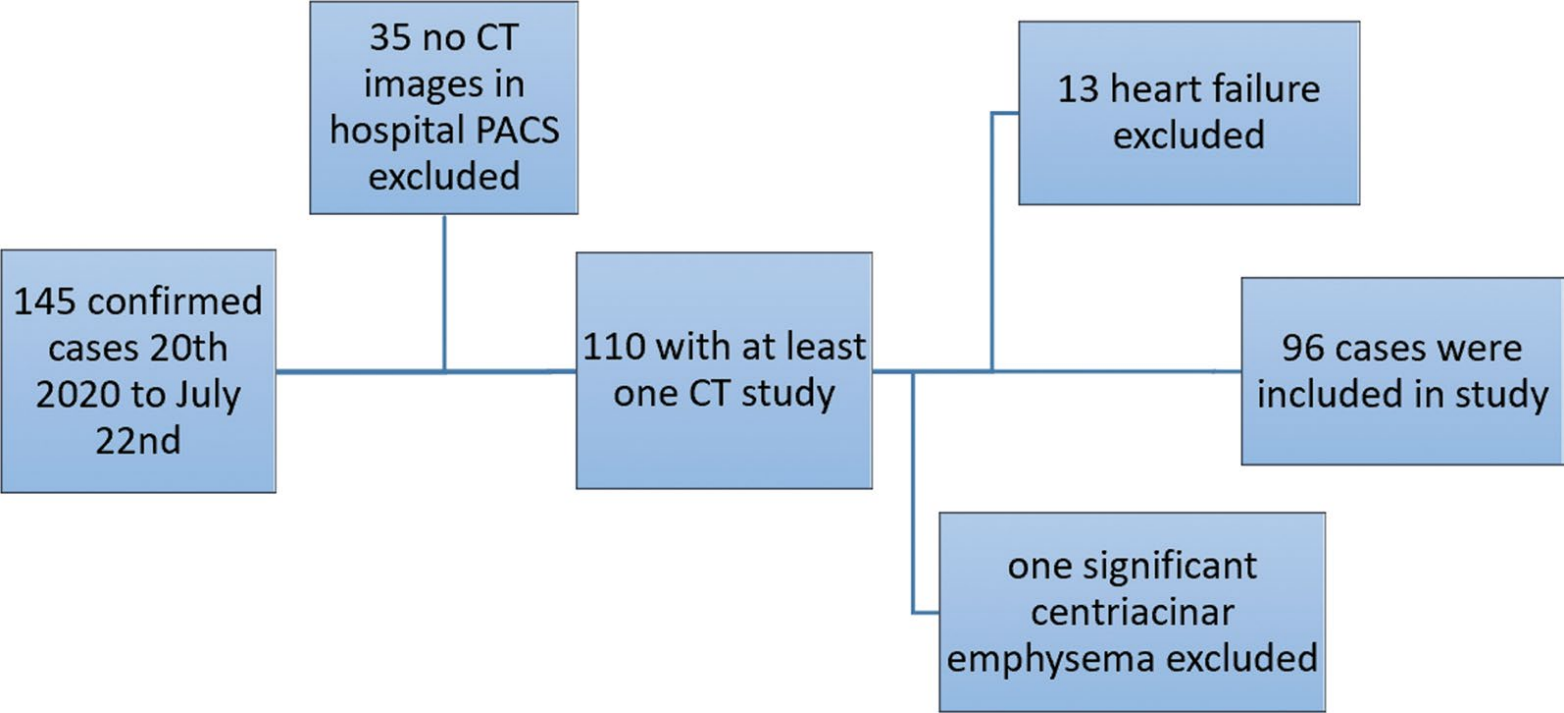
Flowchart for patient selection

Among COVID-19 patients who referred to our hospital from February 20, 2020, to July 22, there were 145 confirmed cases. Of these patients, 110 have had at least one CT scan record in the hospital PACS. After reviewing the first CT images, 14 patients with cardiopulmonary comorbidity were excluded, consisting of 13 patients with significant heart failure and one patient with significant centrilobular emphysema. Ninety-six patients were included in the study. Patient selection process is summarized in Fig. 1.

The demographic data of the included patients, number of moderate, severe and critical patients at presentation and at peak disease severity and the number who died are summarized in Table 3.

**Table 3:** Patients’ demographics and distribution of disease severity at presentation and at peak disease severity

	Number (Male/Female)	Mean age ± SD
Total	96 (57/39)	63.6 ± 17.4
Moderate disease at presentation	41 (25/16)	57.3 ± 18.9
Severe disease at presentation	53 (31/22)	68.2 ± 14.9
Critical disease at presentation	2 (1/1)	71.5 ± 6.4
Moderate disease at peak severity	22 (13/9)	52.5 ± 20.1
Severe disease at peak severity	31 (17/14)	62 ± 16.8
Critical disease at peak severity	43 (27/16)	70.4 ± 12.9
Discharged	56 (32/24)	59 ± 18.6
Deceased	40 (25/15)	70 ± 13.3

All 96 patients underwent initial thoracic CT scan within first 24 h of admission, on average 4 ± 3.4 days (range 0–19 days, median 3 days) after the onset of symptoms.

Interrater reliabilities between two raters for CTSSs 1–7 calculated as ICCs, as well as related inference, are presented in Table 4. All CTSSs showed good interrater reliability as ICC = 0.764–0.837. CTSS2 and CTSS7 showed the largest values (0.837 and 0.834, respectively).

**Table 4:** Interrater reliability between the two radiologists and related inference

CT Severity Score	Intraclass correlation	Inference about interrater reliability
CTSS1	0.783	Good
CTSS2	0.837	Good
CTSS3	0.764	Good
CTSS4	0.778	Good
CTSS5	0.773	Good
CTSS6	0.834	Good
CTSS7	0.784	Good

**Fig. 2:**
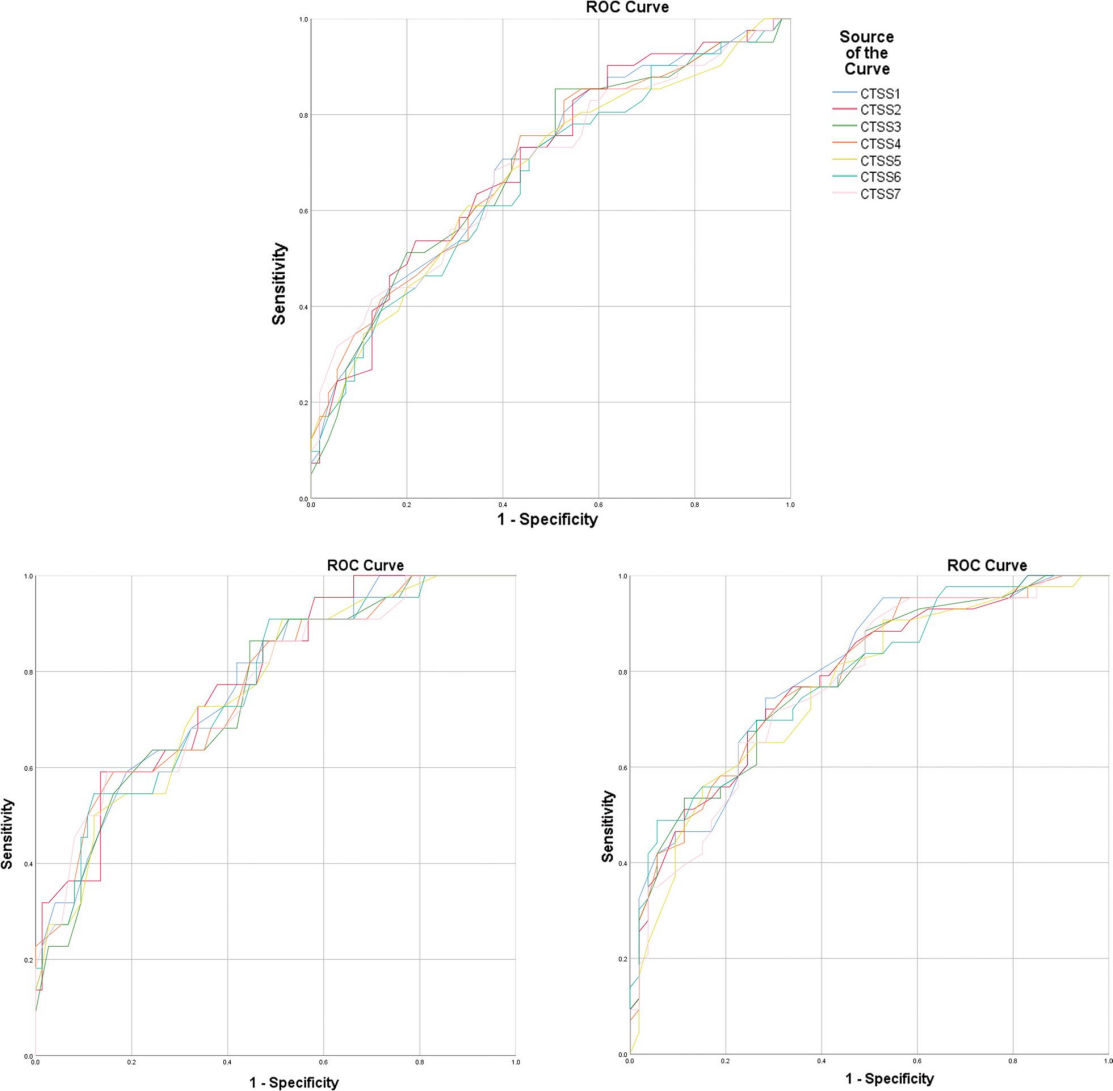
Top: ROC curves plotted for different average CTSSs at initial CT scan discriminating moderate from severe/critical disease at the time of hospital admission; bottom left: ROC curve for the same severity discrimination in the most severe disease period; and bottom right: ROC curve for discrimination of moderate/severe from critical disease in the most sever disease period

AUC for ROC curves for discriminating patients in moderate from severe/critical group at the time of admission as well as related inference, threshold, sensitivity and specificity for each CTSS is presented in Table 5 (upper set). Only three CTSSs, namely CTSS1, CTSS2 and CTSS4, showed sufficient AUCs to be useful in triage (AUC = 0.70). The sum of sensitivity and specificity for the best threshold values was 131–132% for the mentioned CTSSs. Corresponding ROC curves are shown in Fig. 2 (top). Pairwise comparison of AUCs of these ROC curves by z test showed that there is no significant difference between them.

**Table 5:** AUC, confidence interval, related inference, best threshold and related sensitivity and specificity for ROC curves about different CTSSs about diagnosis of severe/critical group at presentation and at peak disease severity and also for diagnosis of critical disease at peak severity

	Average CTSS	AUC for ROC curve	95% confidence interval	Inference about AUC	Best threshold	Sensitivity/specificity %
Diagnosis of severe/critical patients at presentation	CTSS1	0.70	0.59–0.80	Acceptable	11	60/71
	CTSS2	0.70	0.60–0.81	Acceptable	15	78/54
	CTSS3	0.69	0.58–0.80	Unsatisfactory	12	49/85
	CTSS4	0.70	0.59–0.80	Acceptable	14.5	56/76
	CTSS5	0.68	0.57–0.79	Unsatisfactory	13.5	67/61
	CTSS6	0.67	0.56–0.78	Unsatisfactory	24.5	53/73
	CTSS7	0.69	0.58–0.80	Unsatisfactory	16	62/68
Diagnosis of severe/critical patients at peak disease severity	CTSS1	0.78	0.67–0.88	Acceptable	7.5	81/59
	CTSS2	0.78	0.68–0.89	Acceptable	13	87/59
	CTSS3	0.76	0.65–0.87	Acceptable	9.5	55/86
	CTSS4	0.77	0.66–0.88	Acceptable	10	84/59
	CTSS5	0.76	0.65–0.87	Acceptable	16	92/49
	CTSS6	0.77	0.65–0.88	Acceptable	15.5	88/55
	CTSS7	0.77	0.65–0.88	Acceptable	11.5	85/59
Diagnosis of critical patients at peak disease severity	CTSS1	0.79	0.70–0.88	Acceptable	10.5	74/72
	CTSS2	0.78	0.69–0.87	Acceptable	19	72/72
	CTSS3	0.78	0.69–0.87	Acceptable	9.5	70/74
	CTSS4	0.79	0.70–0.88	Acceptable	13.5	74/68
	CTSS5	0.76	0.67–0.86	Acceptable	16.5	56/85
	CTSS6	0.79	0.70–0.88	Acceptable	22.5	70/74
	CTSS7	0.77	0.68–0.86	Acceptable	17.5	65/77

ROC curves AUCs for predicting severe/critical disease at the time of peak disease severity as well as related inference, threshold, sensitivity and specificity for each CTSS are presented in Table 5 (middle set). All CTSSs showed acceptable AUCs (0.76–0.78). The sum of sensitivity and specificity for the best thresholds was 140–146% for different CTSSs. Corresponding ROC curves are shown in Fig. 2 (bottom left). Pairwise comparison of AUCs of these ROC curves showed that there is no significant difference between them.

AUC for ROC curves for predicting critical disease at the time of peak disease severity as well as related inference, threshold, sensitivity and specificity for each CTSS is also presented in Table 5 (lower set). All CTSSs showed acceptable AUCs (0.77–0.79). The sum of sensitivity and specificity for the best thresholds for such diagnosis was 141–146% for different CTSSs. Corresponding ROC curves are shown in Fig. 2 (bottom right). Pairwise comparison of AUCs of these ROC curves showed that there is significant difference only in CTSS1-CTSS5, CTSS4-CTSS5, CTSS1-CTSS7 and CTSS4-CTSS7 pairs (*p* value = 0.04 for all four pairs) and no significant difference was present in the other pairs.

## Discussion

Many researchers have used CTSS as a disease quantifying tool in COVID-19 [1–10]. Some of them evaluated CTSS by ROC curve AUC, sensitivity, specificity and other indices of test performance and also by interrater reliability [6, 10, 11]. To the best of our knowledge, 6 types of CTSS have been proposed and we propose another one (CTSS5). We evaluated 7 CTSS types for their performance in triage and prognostication and also interrater reliability.

Because RT-PCR rarely if ever had been ordered for patients with mild symptoms in our institution, due to lack of resources, our cohort is composed of more severely affected patients in comparison with the other studies [3–10] with much higher mortality rate (42%). As most of other mentioned studies [3–5, 8–10], men were more frequent in our cohort than women (57 vs. 39). This may indicate that women are affected less, probably because of estrogen protective effect [22] or possibly they less frequently seek medical assistance.

**Fig. 3:**
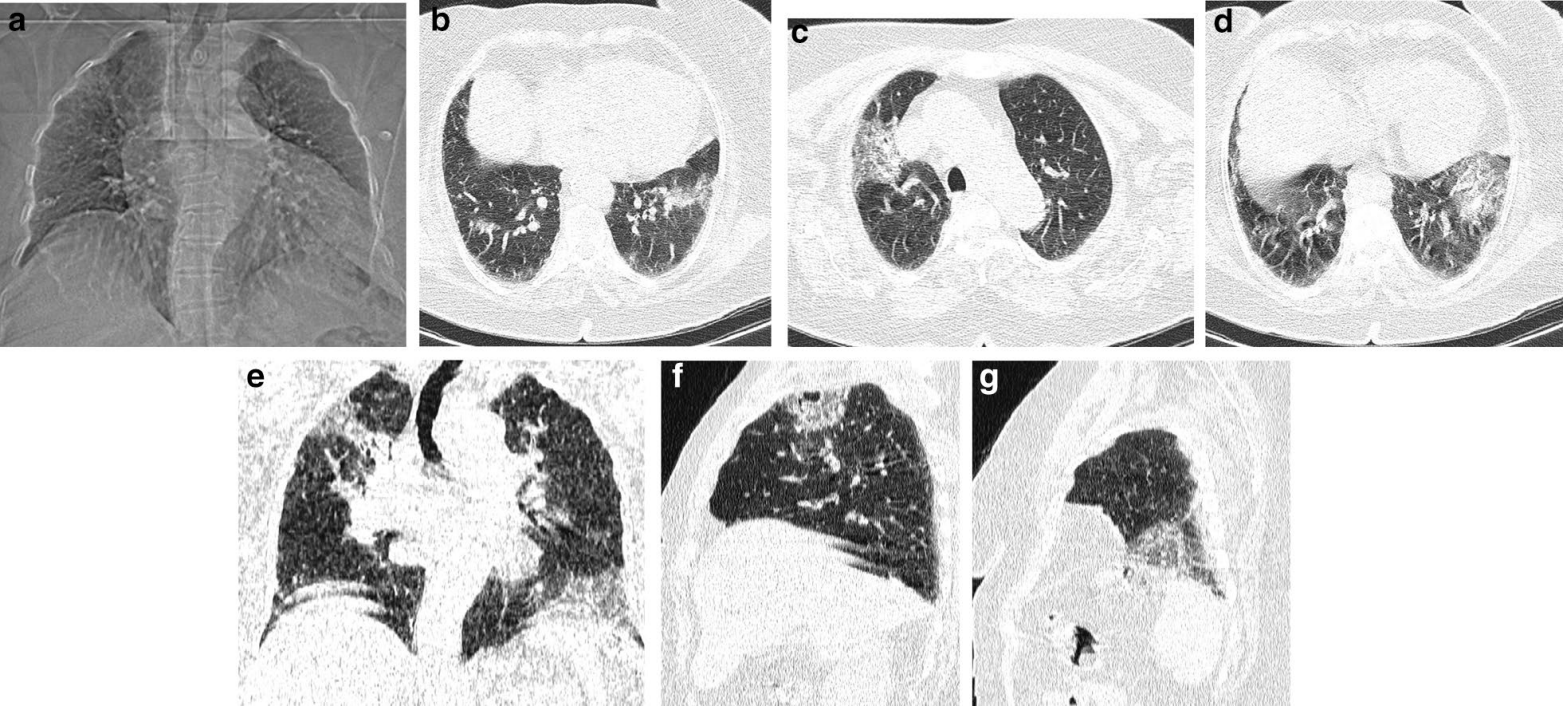
A 78-year-old, non-diabetic lady with heart failure, suggested by a large heart on topogram image (CT ratio > 60%) (**A**) and large intrapulmonary arteries (broncho-arterial ratio < 0.5) (**B**), showed mild lung involvement in her first-day in-hospital CT (**C**–**G**) with CTSS1 = 3/24, CTSS2 = 5/48, CTSS3 = 3/20, CTSS4 = 5/25, CTSS5 = 7/20, CTSS6 = 7/40, CTSS7 = 5/30. After 7 days in intensive care unit (ICU) (severe disease on triage), she got intubated and died after another day (critical disease on prognostication)

There are many comorbidities which may aggravate COVID-19, for example, hypertension, obesity, diabetes, active cancer, chemotherapy, solid organ transplant, chronic kidney disease and immunosuppressive therapy [23]. Most of these comorbidities including hypertension result in disturbances in immune system [23] which may present as more extensive inflammation leading to higher scores on CT images. Regarding CT severity quantification, two other comorbidities are of special importance: heart failure and preexisting lung disease, because they may lead to more severe disease and higher mortality rate without increasing the extent of COVID-19 lung involvement on CT. Considering the whole COVID-19 patient population, heart failure is a major risk factor for in-hospital mortality [24, 25] with odds ratio of 3.46 reported in a systematic review [23]. Preexisting respiratory disease has also a major impact on the COVID-19 mortality with a reported adjusted odds ratio of 1.36 in a study [26]. *Consequently, it is a good practice to place patients with heart failure or preexisting significant pulmonary disease in the high-risk group without any judgment upon their CTSS.* We regarded heart failure and significant preexisting respiratory disease as confounders and those patient with evidence of these diseases were excluded from data analysis. A case of heart failure (excluded patient) with mild lung involvement with severe disease at hospital admission and critical outcome is presented in Fig. 3. It depicts how a heart failure patient with mildly affected lung by COVID-19 may show severe disease at presentation and eventually show critical disease. That is why we excluded heart failure patients from data analysis.

Our results showed good interrater reliability between two radiologists for all CTSSs (ICC = 0.764–0.837). The best ICCs were for CTSS2 and CTSS6, the two requiring more numerous segmentations. In this regard, our proposed CTSS7 stands in the third place. *Therefore, it is wise to use CTSS2 or CTSS6 if a later follow-up by CT is contemplated or if the scores are going to be used in an analytical study.*

We failed to reproduce the brilliant interrater reliability reported in the earlier studies as ICC for CTSS3 had been reported to be 0.976 [6], but we computed 0.764. ICC for CTSS6 had been reported 0.936 [10], but we computed 0.834. The difference between previously reported ICC values and our reported ICCs can be due to two reasons. First, overall, is more severe disease in our cohort, making scoring process more complex, and second and more important is that we decided to compute ICCs based on 2-way random model, single measurement form and absolute agreement type (ICC_1,1_ with absolute agreement) which produces the lowest ICC values, but is the most reliable one among the 10 ICC classes if reproducibility of the test is to be evaluated [18, 19]. For CTSS3, the authors did not mention that what model, form and type of ICC they were reporting [6]; therefore, comparison with our study is not accurate. The same is true for reported CTSS6 ICC [10].

We evaluated discriminatory performance of CTSSs between the two moderate and severe/critical groups for triage. Calculated AUCs ranged 0.67–0.7, and there were only three CTSSs with sufficient ROC curve AUCs to be suitable for clinical implementation in triage of the patients, although they showed borderline value (0.70). They were CTSS1, CTSS2 and CTSS4, and their performance was far from ideal. Again, these results are not compatible with earlier studies; as for CTSS3 the reported AUC for diagnosing severe/critical disease was 0.918 (95% CI 0.962–0.985) and CTSS3 cutoff of 7.5 had 82.6% sensitivity and 100% specificity in diagnosing severe/critical group [6]. Our computed AUC value is 0.69 for AUC which is regarded as unsatisfactory. The same is true for CTSS6 with reported AUC of 0.892 (95% CI 0.814, 0.944) and that CTSS6 cutoff value of 19.5 had 83.3% sensitivity and 94% specificity in diagnosing severe/critical groups [10], but our calculated AUC is 0.67 (CI 0.56–0.78), again unsatisfactory. This discrepancy in results is most probably because of relative low incidence of severe/critical disease in the mentioned studies as their cohort included only about 10% severe/critical disease patients in CTSS3 study [6] and less than 18% in CTSS6 study [10], but in our study the corresponding percentage is 57%. We do not favor a very powerful role for CTSS in triage of patients, although some role still exists, more specifically for CTSS1, CTSS2 and CTSS4. Therefore, *if CTSS is to be used for triage of patients, using CTSS1, CTSS2 or CTSS4 is recommended.*

CTSSs performed better in prognostication than in triage with acceptable AUCs for all the CTSSs both in discriminating moderate from severe/critical group and discriminating moderate/severe from critical group at peak disease severity, as all the related AUCs were acceptable for clinical use with AUCs of 0.76–0.79. *Hence, all the CTSSs are acceptable for prognostication.*

**Fig. 4:**
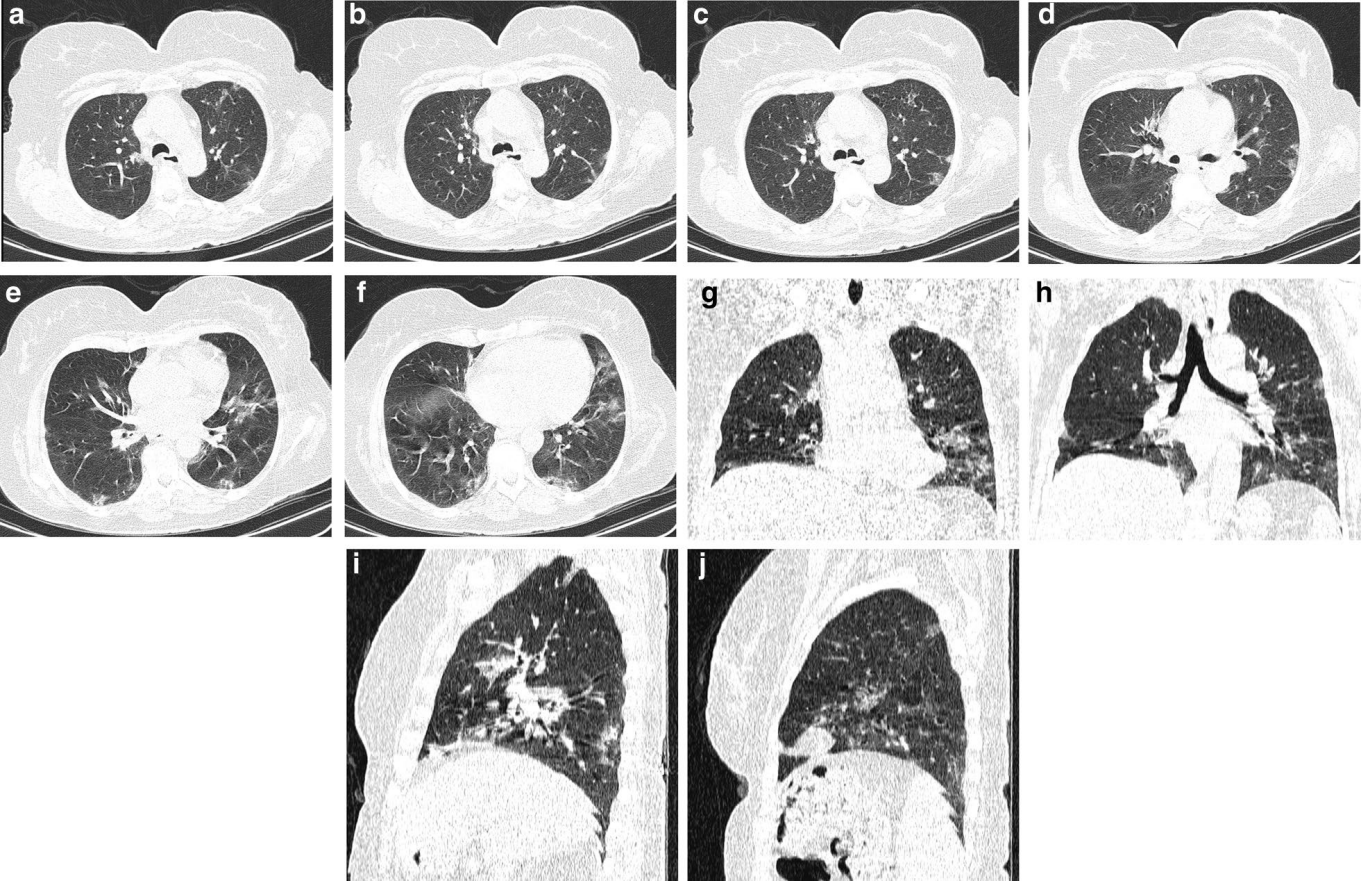
A 62-year-old, non-diabetic lady without any identifiable risk factor showed some lung involvement in her first-day in-hospital CT (**A**–**J**) CTSS1 = 8/24, CTSS2 = 14/48, CTSS3 = 6/20, CTSS4 = 12/25, CTSS5 = 10/20, CTSS6 = 17/40, CTSS7 = 14/30. After admission, she stayed in the ward for 6 days (moderate disease on triage), and then, disease aggravation led to admission in ICU (severe disease on prognostication); she was not intubated and was discharged after staying 7 days in ICU

A case of moderate disease at presentation with progression to severe disease after 6 days is shown in Fig. 4.

Recent reports show results compatible with our study as Hajiahmadi and colleagues reported ROC curve AUC 0.764 for CTSS1 for predicting severe/critical disease in a cohort including 24% severe/critical disease patients [27], while our calculated figure was 0.79. In addition, Aminzadeh and co-workers used a CTSS method similar to our CTSS7 and reported ROC curve AUC of 0.65 for triage of severe/critical patients and 0.76 for predicting critical disease at peak disease severity [28], while our corresponding calculated values for CTSS7 were 0.69 and 0.77, respectively.

Two limitations should be considered: One is the absence of mildly diseased patients in our cohort which was because RT-PCR was not ordered routinely for mildly diseased patients who were not hospitalized. The other one was the absence of long-term follow-up after discharge to evaluate the relation of CTSSs to long-term sequelae of COVID-19.

## Conclusions

- Quantification of lung disease in COVID-19 is a readily available and easy tool to be used in triage and prognostication, but its use is not encouraged in heart failure or chronic respiratory disease patients. These patients are already at high risk of critical disease irrespective of CTSS.
- Those scoring systems requiring more numerous segmentations, namely CTSS2, CTSS6 and CTSS7, show better interrater reliability.
- There is not much difference between different CT severity scoring systems in terms of their AUCs for triage and prognostication.
- CTSS has a limited value in triage, and CTSS1, CTSS2 and CTSS4 showed the best AUCs in this regard in our study.
- All CTSSs show acceptable performance in prognostication.

## Data Availability

The datasets used and/or analyzed during the current study are available from the corresponding author on reasonable request.

## Abbreviations

AUC: Area Under the Curve
CI: Confidence Interval
CTSS: Computed Tomography Severity Score
HIS: Hospital Information System
ICC: Intraclass Correlation Coefficient
PACS: Picture Archiving and Communication System
ROC curve: Receiver Operator Characteristic curve
RT-PCR: Reverse-Transcriptase Polymerase Chain Reaction
Sens.: Sensitivity
Spec.: Specificity
